# Supplementary material for: Intravenous indocyanine green dye is insufficient for robust immune cell labelling in the human retina
Source: PLoS One. 2020 Feb 13;15(2):e0226311. doi: 10.1371/journal.pone.0226311 (PMC7018502; doi:10.1371/journal.pone.0226311)
Supplement: S1 Table — Measured from time of ICG injection. Timings were determined by participant availability and following an amendment, extension to cover a range of longer intervals to maximise the chance of signal detection. (DOCX) [file pone.0226311.s001.docx]

**S1 Table.** **Imaging session and blood sample collection timings for each participant.**

| **ID** | **Imaging Session 1** | **Imaging Session 2** | **Imaging Session 3** | **Imaging Session 4** | **Blood Sample 1** | **Blood Sample 2** |
| --- | --- | --- | --- | --- | --- | --- |
| 1 | 0 to 8 hours | 24 hours | 48 hours | Day 7 | 4 hours | 24 hours |
| 2 | 0 to 8 hours | 24 hours | 48 hours | Day 7 | 4 hours | 24 hours |
| 3 | 0 to 8 hours | 24 hours | 48 hours | Day 7 | 4 hours | 24 hours |
| 4 | 0 to 8 hours | 24 hours | 48 hours | Day 7 | 4 hours | 24 hours |
| 5 | 0 to 8 hours | 24 hours | 48 hours | Day 7 | 2 hours | 24 hours |
| 6 | 0 to 8 hours | 48 hours | Day 7 | Day 9 | 4 hours | Day 7 |
| 7 | 0 to 8 hours | 24 hours | Day 7 | Day 9 | 4 hours | 24 hours |
| 8 | 0 to 8 hours | 24 hours | Day 7 | Day 9 | 2 hours | Day 7 |
| 9 | 0 to 8 hours | 24 hours | Day 7 | Day 9 | 2 hours | 24 hours |
| 10 | 0 to 8 hours | 24 hours | Day 7 | Day 9 | 2 hours | 24 hours |
| 11 | 0 to 8 hours | 24 hours | Day 7 | Day 9 | 2 hours | Day 7 |
| 12 | 0 to 8 hours | 24 hours | Day 7 | Day 9 | 2 hours | 24 hours |
