# Supplementary figures and images for: Intravenous indocyanine green dye is insufficient for robust immune cell labelling in the human retina
Source: PLoS One. 2020 Feb 13;15(2):e0226311. doi: 10.1371/journal.pone.0226311 (PMC7018502; doi:10.1371/journal.pone.0226311)

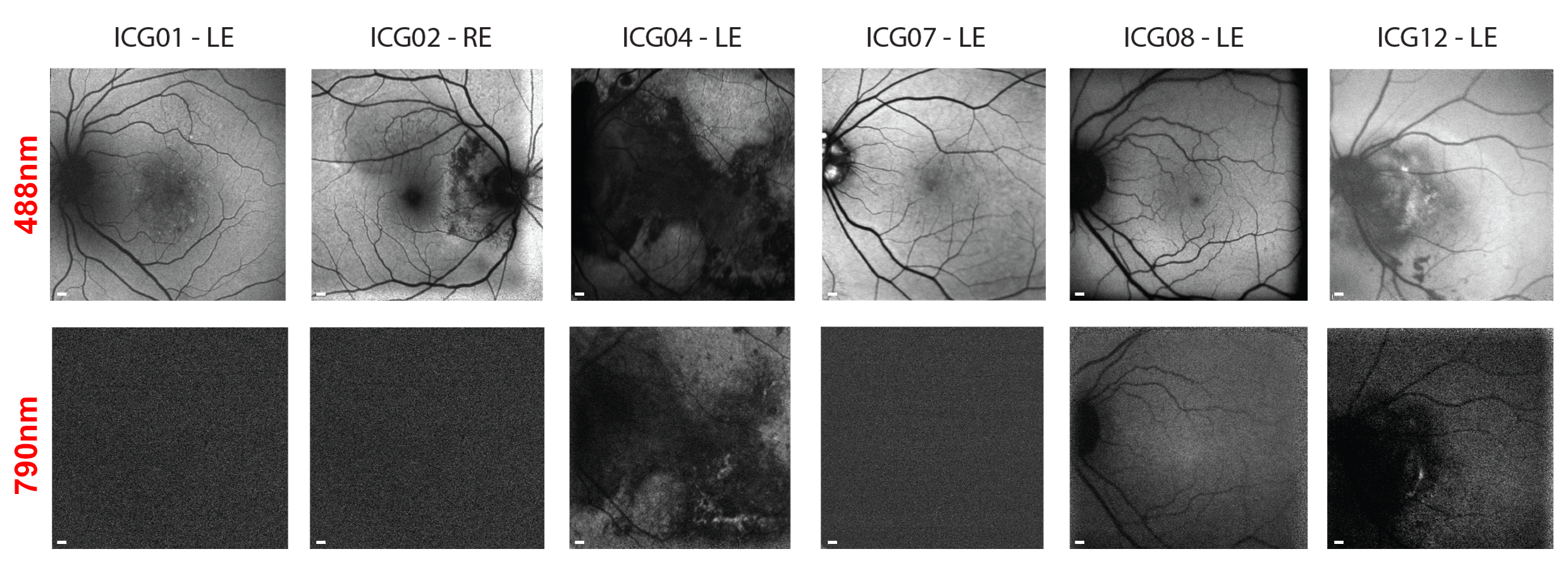

Supplement: S1 Fig — A representative selection of baseline images acquired from patients in the study illustrating weak to minimal infrared autofluorescence prior to ICG administration. Scale bars = 300 μm. (TIF) [file pone.0226311.s002.tif]

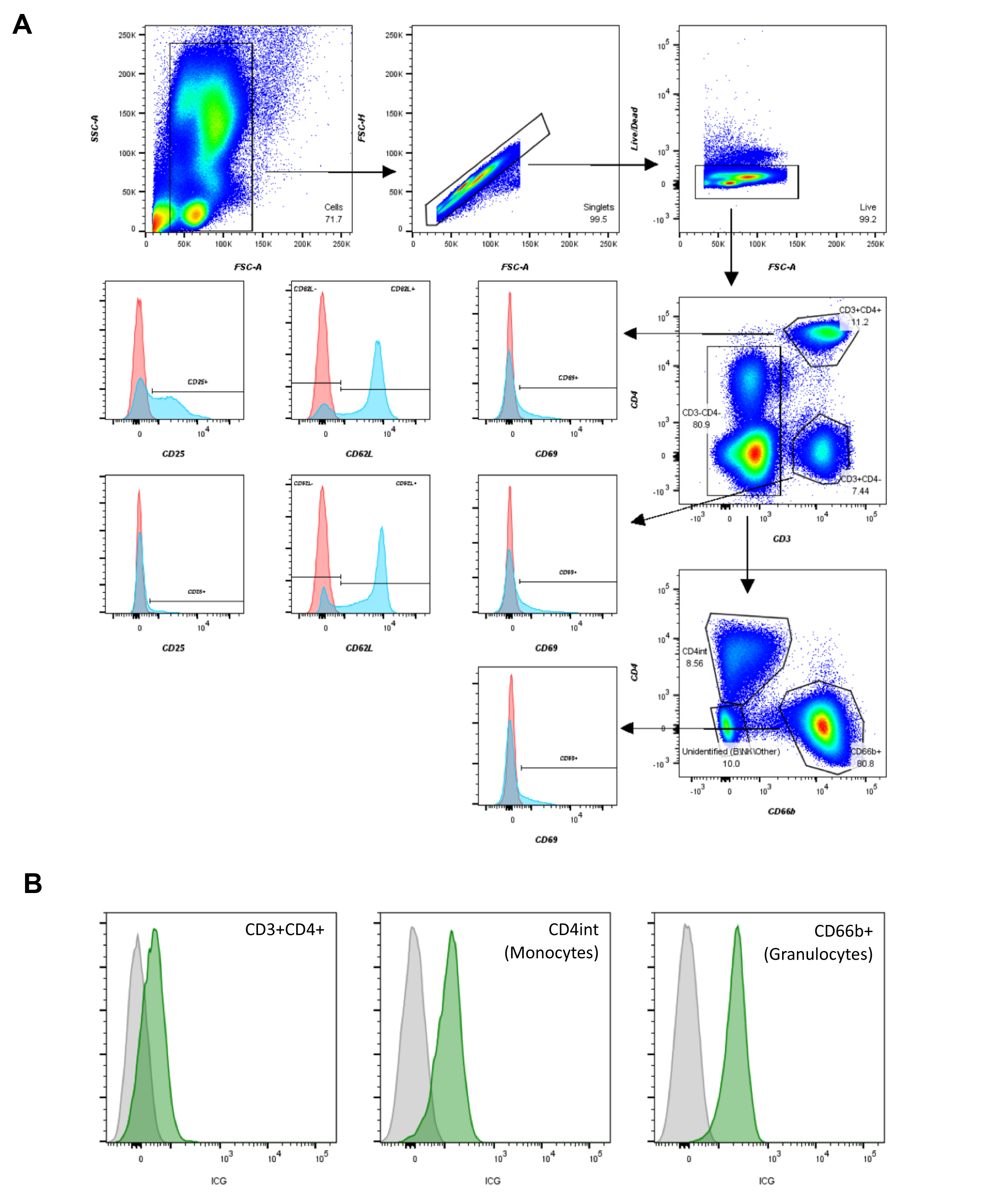

Supplement: S2 Fig — (A) The representative gating strategy used for antibody cocktail #1 (“lymphocyte panel”) is shown. For antibody cocktail #2 (“monocyte panel”), live cells were gated into granulocytes (CD66b+) or monocytes (CD14+ CD66b-, a CD14 FMO was used to assist with drawing the CD14+ gate)). For the histograms (activation markers: CD25, CD62L, and CD69), FMOs are indicated in red, whilst a representative participant sample is indicated in blue. Validation of the activation marker antibodies was performed via whole-blood staining (individually for 24 hours) with phytohaemagglutin P (PHA; at a final concentration of 20 μg/mL), CD3/CD28 activating beads (Dynabeads; 0.63 μL of beads (stock concentration 4.0 * 107 beads/mL) were used), and li popolysaccharide (LPS; at a final concentration of 0.1 ng/mL). (B) Representative histograms from an experiment where whole human blood was stained ex vivo with 6 ug/mL indocyanine green (ICG) for 2 hours (green), as compared to an ICG FMO sample (grey). The staining is less bright than has been observed with cell lines and the participant data, possibly due to the abundance of erythrocytes (as compared-to cell cultures), lower ICG concentration, and/or shorter staining time. (TIF) [file pone.0226311.s003.tif]

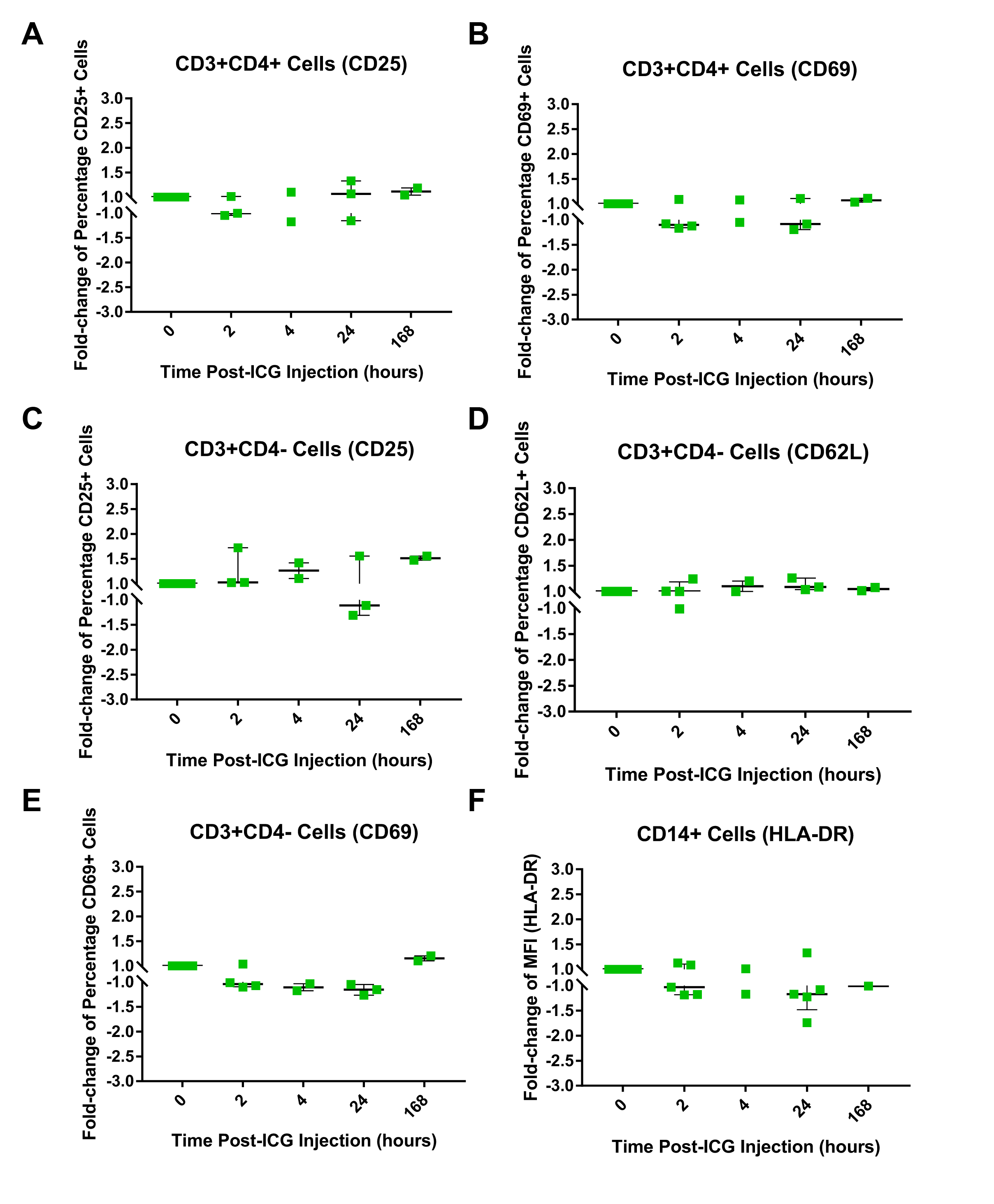

Supplement: S3 Fig — (A–B) Scatterplots for CD3+CD4+ cells show changes in CD25 and CD69, (C–E) for CD3+CD4- cells show changes in CD25, CD62L, and CD69, and (F) for CD14+ cells (monocytes) show changes in HLA-DR. The y-axis is expressed as fold-change in the percentage of positive cells (CD25, CD62L, CD69) or the fold-change of the MFI (CD80, HLA-DR). Loss of CD62L is associated with activation, whilst expression of CD25 and CD69, and up-regulation of CD80 and HLA-DR associate with activation; statistical tests were not significant (p ≥0.05). The data was acquired using flow cytometry (n = 7). (TIF) [file pone.0226311.s004.tif]
