# Supplementary material for: Intravenous indocyanine green dye is insufficient for robust immune cell labelling in the human retina
Source: PLoS One. 2020 Feb 13;15(2):e0226311. doi: 10.1371/journal.pone.0226311 (PMC7018502; doi:10.1371/journal.pone.0226311)
Supplement: Attachment — Submitted filename: Response to Reviewers.docx [file pone.0226311.s005.docx]

**Dr. Colin Chu**

Academic Unit of Ophthalmology

Translational Health Sciences

University of Bristol

Bristol, BS8 1TD

United Kingdom

Tel: +44(0)1173312032

Fax: +44(0)1173312032

[colin.chu@bristol.ac.uk](mailto:colin.chu@bristol.ac.uk)

21^st^ January 2020

Dear Reviewers,

**Re: PONE-D-19-32243: Intravenous indocyanine green dye is insufficient for robust immune cell labelling in the human retina**

Thank you for your comments and suggestions in relation to the manuscript. We have addressed their requests and those from the editorial team as detailed below and hope that the manuscript is now acceptable for publication.

Yours sincerely,

Colin Chu

**Reviewer 1**

The manuscript follows up on a previously published paper from the same group that used ICG imaging to detect retinal immune cells in mice. Despite its mostly negative findings that ICG labeling as it stands now is insufficient for robust immune cell labeling in the human retina, the paper is principally very interesting and important. However, the major problem of the study is the limited number of enrolled patients (n=12) and the mixed disease population (nvAMD, Uveitis, CSR). To come to a robust conclusion at least for one disease entity, the authors should limit themselves to one major disease group (e.g. nvAMD under different treatment regimens) and increase the sample number. It is likely that the number of subjects that shows ICG signals in the retinal will then be increased. Also, a correlation of ICG signals with treatment could be interesting, as it is well known that anti-VEGF /anti-PGF treatment may influence retinal immune cell homeostasis.

- We thank the reviewer for their input and suggestions. We agree with the points and think that careful stratification would be likely to maximize the effect size and proportion of those seen with potential ICG labelling in the retina. It would also be very interesting to isolate and study the effects on VEGF blockade.
- Unfortunately, as an exploratory study we were unable to recruit more than 12 patients due to both limited funding from the grant obtained and limits on our ethics approval. Both have now completed and we are unable to recruit any further patients. The reason for the mixed patient population was due to the uncertainty of which disease might have activated immune cells trafficking into the retina, as well as extrapolating from data in the mouse, where potential leakage of subretinal fluid might allow ICG accumulation to label local cells. This is mentioned in the methods section (lines 92–94 & 116 of the revised manuscript). Ideally all recruited patients would have been selected for active panuveitis, but these were a rare population that we would struggle to recruit in sufficient numbers to complete the study, unlike with nvAMD populations.
- We will take note of these suggestions for any subsequent study, which would probably involve either a significant dose increase or more likely, an altered route of administration via subcutaneous or oral formulation routes. We feel however it is important to report the results of this study with these known caveats to inform continuing discussions in the research community.

**Reviewer 2**

Reviewer #2: In the manuscript, entitled:” Intravenous indocyanine green dye is insufficient for robust immune cell labelling in the human retina”, the authors took effort to determine if immune cell infiltration could be detected by routine clinical imagining techniques. Having such an opportunity would help to better understand time course and pathology of ocular inflammation in different eye diseases. This could also be a valuable tool to track treatment related to inflammation as well as to complications related to a leaky blood retina barrier. Unfortunately, only 1 patient demonstrates ICG labeled cells in the blood and in the retina. The authors mention that the low success rate may be due to the low dosage of systemic ICG used for ophthalmic imaging and that increasing the dose might lead to better labelling. The manuscript is interesting, very well written, technically correct and will hopefully pave the way for follow up studies. I have only a few comments, listed below, which should be addressed before I could recommend this manuscript for publication in Plos One.

- We thank the reviewer for their evaluation and supportive comments.

Line 29: “Matched peripheral blood samples…..” It is not quite clear why those samples were analyzed. Please add a half sentence to explain that this experiment was done to determine if ICG labels immune cells.

- We have modified the abstract to more clearly explain the purpose of the blood samples was to detect systemic blood labelling, that might occur as a mechanism resulting in retinal infiltration of ICG-positive cells. We also used these to ensure ICG did not cause overt immune cell activation that would preclude wider application of this strategy (see lines 137–8 of the revised manuscript).

Line 209, Result section: Adding headlines might provide more guidance through the section and help improve the structure of the manuscript.

- Sub-headings have been added to enhance the clarity of the manuscript.

Line 220, Table1: Is there an explanation why patient 1 has only received 0.08 mg/kg ICG? Please also introduce all abbreviations (LE, BE, RE, etc) in the legend with their first occurrence.

- Patients 1 and 2 received low standard doses of ICG used for angiographic imaging in the clinical centre. As these initial two results was negative, the dose was escalated (in-line with our ethical approval) to increase the probability that immune cell labelling could be observed. This was noted in the main text (lines 217–8), but for clarification a note has been added to Table 1 legend of “*Note total ICG dose injected was increased to a fixed maximal 25mg from participant 3 onwards.” The order of abbreviations has also been corrected.

Line 270, Figure 3 legend: The authors state that subject 3 shows ICG positive cell labeling in PBMCs and that ICG positive cells can be found in the retinal parenchyma. However, they fail to explain why there is no ICG signal present in the vasculature. Further explanation of this could prove informative.

- All images shown in Figure 3 were obtained over 24 hours after ICG injection, which is known to have a short half-life of minutes in the circulation, such it is no longer present in the vasculature in a detectable amount in these images. This agrees with other studies that have recently investigated ICG. We have amended the manuscript and added new citations to reflect this (lines 277–9).

Line 463, S1 Figure legends: The figure legends don’t line up with the images and parts of the graphs in S2 are missing, at least in my copy.

- We apologise for an error in image upload, by which the order of display seems to have been lost. We will re-upload the supplementary figures and verify their layout in the final document.
